# CNN-based flow control device modelling on aerodynamic airfoils

**DOI:** 10.1038/s41598-022-12157-w

**Published:** 2022-05-17

**Authors:** Koldo Portal-Porras, Unai Fernandez-Gamiz, Ekaitz Zulueta, Alejandro Ballesteros-Coll, Asier Zulueta

**Affiliations:** 1 Nuclear Engineering and Fluid Mechanics Department, University of the Basque Country, UPV/EHU, Nieves Cano 12, Vitoria-Gasteiz, 01006 Araba, Spain; 2 System Engineering and Automation Control Department, University of the Basque Country, UPV/EHU, Nieves Cano 12, Vitoria-Gasteiz, 01006 Araba, Spain

**Keywords:** Engineering, Mathematics and computing, Physics

## Abstract

Wind energy has become an important source of electricity generation, with the aim of achieving a cleaner and more sustainable energy model. However, wind turbine performance improvement is required to compete with conventional energy resources. To achieve this improvement, flow control devices are implemented on airfoils. Computational fluid dynamics (CFD) simulations are the most popular method for analyzing this kind of devices, but in recent years, with the growth of Artificial Intelligence, predicting flow characteristics using neural networks is becoming increasingly popular. In this work, 158 different CFD simulations of a DU91W(2)250 airfoil are conducted, with two different flow control devices, rotating microtabs and Gurney flaps, added on its Trailing Edge (TE). These flow control devices are implemented by using the cell-set meshing technique. These simulations are used to train and test a Convolutional Neural Network (CNN) for velocity and pressure field prediction and another CNN for aerodynamic coefficient prediction. The results show that the proposed CNN for field prediction is able to accurately predict the main characteristics of the flow around the flow control device, showing very slight errors. Regarding the aerodynamic coefficients, the proposed CNN is also capable to predict them reliably, being able to properly predict both the trend and the values. In comparison with CFD simulations, the use of the CNNs reduces the computational time in four orders of magnitude.

## Introduction

In recent years, with the aim of achieving a cleaner and more sustainable energy model, wind energy has become an important source of electricity generation. Even so, an improvement in wind turbine performance is still required in order to compete with conventional energy sources in terms of energy production and associated costs. To solve this challenge, the implementation of both active, such as rotating microtabs, and passive, such as Gurney flaps, flow control devices is a widely used solution. Aramendia et al.^1,2^ extensively reviewed the available active and passive flow control devices for wind turbines.

Simulations by means of CFD tools are the most popular method for analyzing and optimizing the performance of airfoils and their flow control devices. Many authors have studied several different flow control devices applied on airfoils by means of CFD. For example, Fernandez-Gamiz et al.^3^ and Aramendia et al.^4,5^ performed parametric studies to analyze the effects of the implementation of passive microtabs and Gurney flaps, respectively, on the TE of the DU91W(2)250 airfoil.

In some cases, CFD simulations can be very demanding in terms of computational time and resources, especially when several simulations are necessary to optimize a flow control device or accurate turbulence modeling is required. For this reason, many authors have used alternative meshing models to reduce simulation time. Among these models, the cell-set model, introduced by Ballesteros-Coll et al.^6^ can be highlighted. In that work, different Gurney flaps were added to the TE of a DU91W(2)250 airfoil by means of the cell-set model. In further studies, Ballesteros-Coll et al.^7,8^ implemented microtabs and rotating microtabs on the same airfoil. Other authors, such as Portal-Porras et al.^9,10^ used this meshing technique to model the performance of three-dimensional Vortex Generators (VG) on a flat plate. All of them showed good agreements between the cell-set model, the fully-resolved model and the experimental data. Therefore, this meshing model is considered suitable for this kind of problems.

Despite the accurate predictions that can be obtained through CFD simulations, the increase in the computing speed of computers and the growth of Artificial Intelligence (AI) have led to an increasing number of studies in which Deep Learning (DL) techniques are used for flow prediction, obtaining a significant reduction in terms of computing time. For example, Ye et al.^11^ proposed a Convolutional Neural Network (CNN) to predict the pressure distributions around a cylinder based on the velocity field on its wake behind, Guo et al.^12^ and Ribeiro et al.^13^ designed different CNNs for two- and three-dimensional laminar flow prediction, Portal-Porras et al.^14^ used a CNN to predict turbulent flows on a channel, and Abucide-Armas et al.^15^ proposed a data augmentation technique to improve the predictions of the CNN proposed by Ribeiro et al.^13^ for unsteady turbulent flows.

Regarding airfoils, several machine learning algorithms have been proposed to study their performance. Initially, the most commonly used architecture was the Multi-Layer Perceptron (MLP) learning architecture. For example, Sekar et al.^16^ presented a two-step-consistent approach to predict the flow fields over an airfoil using deep learning techniques. In the first step the airfoil is parametrized into 16 airfoil parameters by means of a CNN, and in the second step a deep MLP network is used to predict the flow fields over airfoils. Even so, this architecture is not specifically designed to exploit spatial and temporal correlation that are intrinsic in many real-world problems. For this reason, the vast majority of current studies similar to the present one use other types networks, being the most common ones the CNNs. Yilmaz and German^17^ studied the airfoil pressure coefficient predictions provided by a CNN by varying its parameters, providing an initial approximation for a suitable CNN design. Thuerey et al.^18^ proposed a CNN to approximate the velocity and pressure fields obtained by Reynolds-Averaged Navier-Stokes (RANS)-based Spalart-Allmaras^19^ turbulence model on airfoils.

For the prediction of aerodynamic coefficients, there is a broader variety of architectures used, since these studies offer greater design flexibility than those of the fields. However, as stated by Zhang et al.^20^, although other networks can provide similar predictions, the CNN is the one that offers the greatest generalization capacity, allowing different geometries to be introduced as input in the network in a simple way. For example, in that study the lift coefficient of different airfoils are predicted with various network architectures. Chen et al.^21^ used a CNN to predict the drag ($${C}_{D})$$ and lift ($${C}_{L}$$) coefficients of different airfoils.

Regarding flow control devices, there are some studies in which deep learning techniques are used for a better understanding of the behavior of flow control elements in airfoils. For example, Rodriguez-Eguia et al.^22^ and Aramendia et al.^5^ used ANNs to predict the aerodynamic coefficients of an airfoil with flaps and Gurney flaps, respectively. However, in these studies the parameters are not predicted directly from the geometry and boundary conditions. Therefore, there are no deep learning studies applied to CFD in which the behavior of flow control elements is analyzed.

The present paper aims to evaluate the possibility of analyzing flow control elements applied to airfoils by means of deep learning techniques. For this purpose, velocity and pressure fields around different Gurney flaps and rotating microtabs implemented on the TE of the DU91W(2)250 airfoil by means of a CNN are predicted. In addition, a variation of the CNN is proposed to predict the ${C}_{D}$ and ${C}_{L}$ coefficients of the studied airfoil with each flow control device.

The remainder of the manuscript is structured as follows: “Methodology” provides an explanation of the methodology followed for preparing and running the CFD simulations and designing and training the proposed CNNs; “Results and discussion” shows qualitative and quantitative comparisons between the CFD results and CNN predictions; and “Conclusion” explains the conclusions reached from this study.

## Methodology

### CFD setup

With the purpose of obtaining data for training, validating and testing the proposed neural network, 158 CFD simulations of the DU91W(2)250 airfoil were conducted, each one under different conditions in terms of flow control device geometry and angle of attack (AoA). The selected profile, DU91W(2)250, is a profile extracted from a broadly referenced NREL horizontal axis wind turbine (HAWT), as stated by Jonkman et al.^23^. Star-CCM+ v2019.1^24^ commercial code was used to run these simulations.

**Figure 1:**
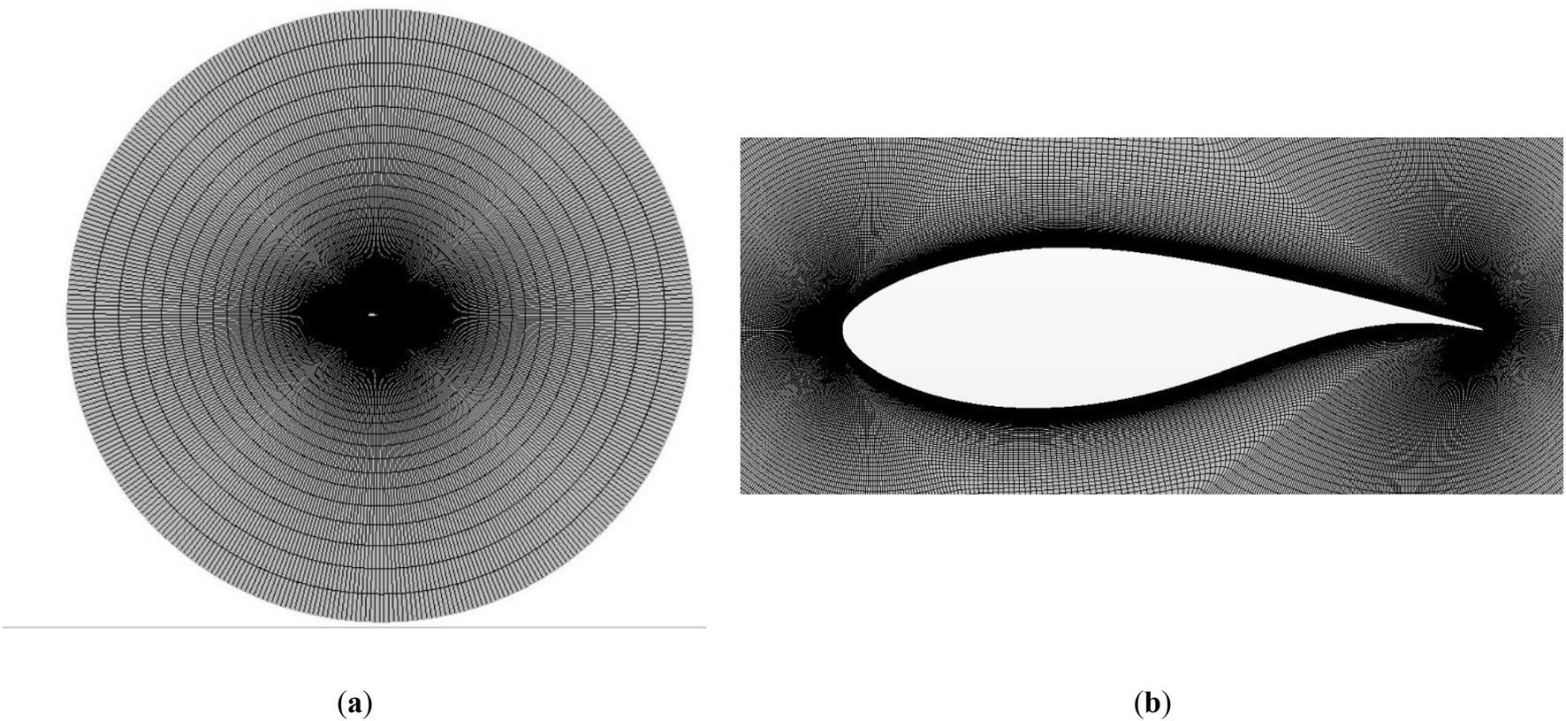
Structured mesh generated around the airfoil: (**a**) general view; (**b**) detailed view.

To perform the mentioned simulations, a two-dimensional structured O-mesh was generated, with the studied airfoil on its center. Following the study of Sørensen et al.^25^, the radius of this mesh (R) was defined as a function of the chord length (c) of the airfoil, $R=32\cdot c$. Therefore, as the chord length of the DU91W250 airfoil is equal to 1 m, R was set at 32 m. This mesh consists of around 207,000 cells. Non-slip conditions were assigned to the contour of the airfoil, and the first cell height ($\Delta z$) normalized with the chord length of the airfoil was set at $\Delta z/c =1.35\cdot {10}^{-6}$. On previous works, Fernandez-Gamiz et al.^3^ studied the mesh dependency of the used mesh, and showed a dependency below 4% for the calculation of ${C}_{L}$ and drag ${C}_{D}$ coefficients. Figure 1a provides a general view of the mesh, and Fig. 1b, a close-up view of the near-airfoil region.

In order to validate the results obtained with this mesh, the obtained results are compared with the experimental data results from the LM Low Speed Wind Tunnel obtained by Timmer^26^. For this comparison, the clean airfoil with different angles of attack is considered.

**Figure 2:**
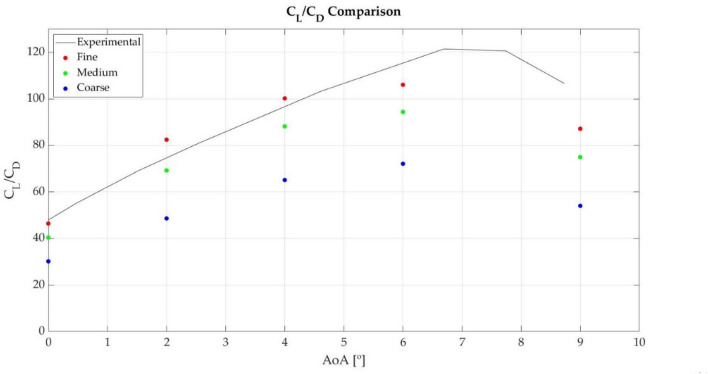
Lift-to-drag ratio comparison of the three generated meshes and the experimental data obtained by Timmer^26^.

In addition, a mesh convergence study is performed considering the lift-to-drag ratio, in order to verify that the numerical solution is independent of the mesh resolution. With this purpose, the General Richardson Extrapolation^27^ method is used. To apply this method, three different meshes are generated, with a mesh refinement equal to 2. The fine mesh consists of around 207,000 cells, the medium mesh of around 103,000 cells and the coarse mesh of around 52,000 cells. Table 1 summarizes the results obtained for the grid convergence study, were RE is the solution of the Richardson Extrapolation, p the order of accuracy and R convergence condition. As the obtained R values are between 0 and 1, the solution is within the asymptotic range of convergence for all the tested angles of attack. Figure 2 compares the CFD results obtained with each mesh and the experimental data. As the results demonstrate, the results obtained with the fine mesh are close to the experimental ones, and the convergence criteria is fulfilled, which means that the mesh is suitable for these simulations.

**Table 1:** General Richardson extrapolation results for the lift-to-drag ratio.

AoA (°)	Mesh			Richardson extrapolation		
	Coarse	Medium	Fine	RE	p	R
0	30.18	42.72	46.44	48	1.75	0.3
2	48.64	75.84	82.44	84.55	2.04	0.24
4	65.12	92.17	100.19	103.56	1.75	0.3
6	72.1	97.55	106.03	110.28	1.58	0.33
9	54.03	80.17	87.14	89.68	1.91	0.27

Two different flow control devices were added to the TE of the airfoil: Gurney flaps and rotating microtabs. A total of 48 different cases with Gurney flaps were considered, with different lengths and angles of attack; and 105 different cases with rotating microtabs, with different lengths (L), orientations (β) and angles of attack. The remaining five cases consist of the airfoil without flow control devices. All the studied cases are summarized in Table 2.

**Table 2:** Analyzed flow control devices and all their configurations.

Flow control device	Length (in % of c)	Orientation	AoA
Clean airfoil	–	–	0**°**, 2**°**, 4**°**, 6**°**, 9**°**
Gurney Flap	0.25, 0.5, 0.75, 1, 1.25, 1.5, 1.75, 2	–	0**°**, 2**°**, 4**°**, 6**°**, 9**°**
Rotating Microtab	1, 1.5, 2	0°, −15°, −30°, −45°, −60°, −75°, −90°	0°, 1**°**, 2**°**, 3**°**, 4**°**, 5**°**

**Figure 3:**
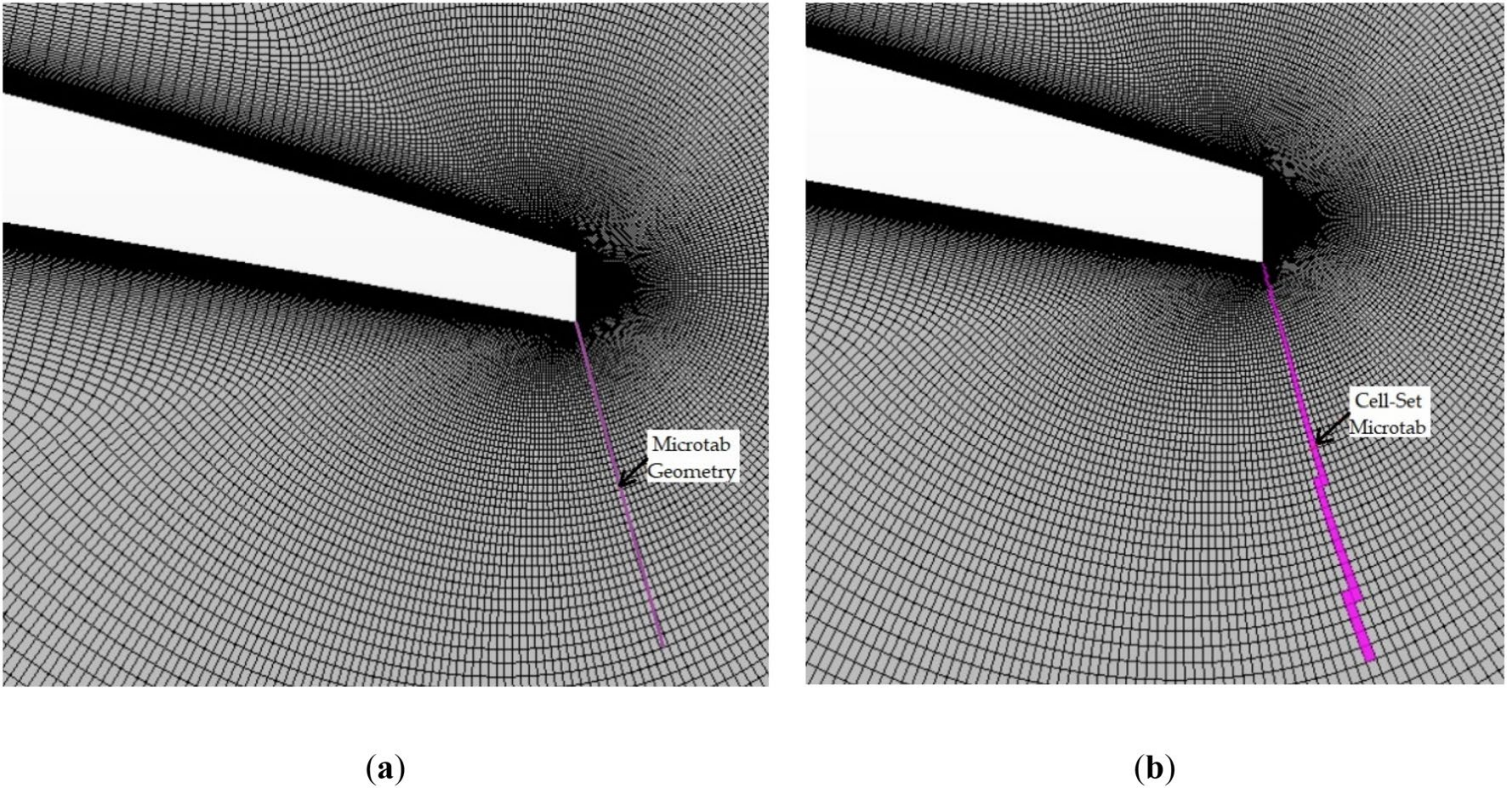
Cell-set implementation for modelling a microtab: (**a**) geometry of the microtab; (**b**) microtab generated using the cell-set model.

These flow control devices were added to the previously-explained mesh by the cell-set model. This modelling technique consists of defining a geometry on an already-generated mesh, and then, splitting this geometry into a new region, and defining it as wall with no-slip conditions. As demonstrated by Ballesteros-Coll et al.^6^, this model is suitable for this kind of problems, since a global relative error of 3.784% of this model in comparison with the fully-resolved model was obtained in that study. Figure 3 illustrates an example of the cell-set model implementation for a rotating microtab.

Regarding the fluid physics, the dynamic viscosity of the air was set at $\mu =1.855\cdot {10}^{-5} {\rm Pa} \cdot {\rm s}$, and the density was set at $\rho =1.2041 \text{kg/}{\text{m}}^{3}$. The freestream velocity of the flow was set at ${U}_{\infty }=30 \text{m/s}$, which means that the Reynolds number (Re) is equal to $2\cdot {10}^{6}$. For turbulence modelling RANS-based k-ω Shear Stress Transport (SST) model by Menter^28^ was chosen, which combines the k-ω model for the near-wall zones and k-ε model for the regions far from the walls. UpWind algorithm was employed for the pressure–velocity coupling and a linear upwind second order scheme was used to discretize the mesh.

### Convolutional neural network

#### Input and output layers

The domain is represented by four different 128 $\times$ 256 layers. The first two layers represent the geometry of the airfoil and the flow control device, and the other two layers represent the velocity components in both directions.

**Figure 4:**
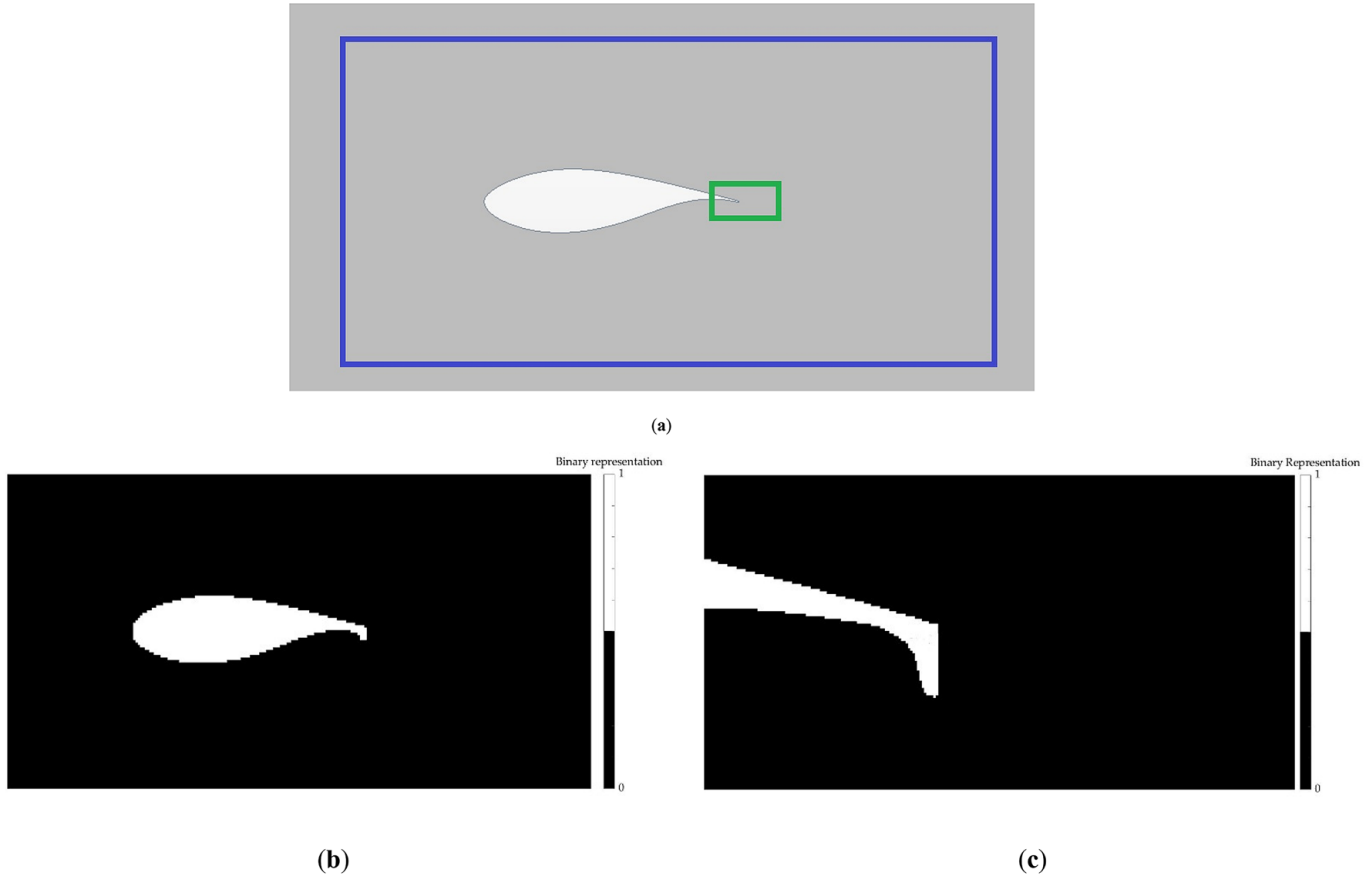
Domain representation layers of a Gurney flap: (**a**) sketch of the area represented by each layer (airfoil overview layer marked in blue and flow control device close-up layer marked in green); (**b**) airfoil overview layer; (**c**) close-up view of the flow control device layer.

The layers describing the domain are generated by means of a binary representation, where the points belonging to the geometry are identified with a 1 and those which do not belong to the geometry are identified with a 0. One of these layers provides an overview of the airfoil, while the other provides a close-up view of the airfoil TE, showing the flow control device in detail. Figure 4a shows the zones represented by each layer, and Fig 4b,c display and example of these two layers.

**Figure 5:**
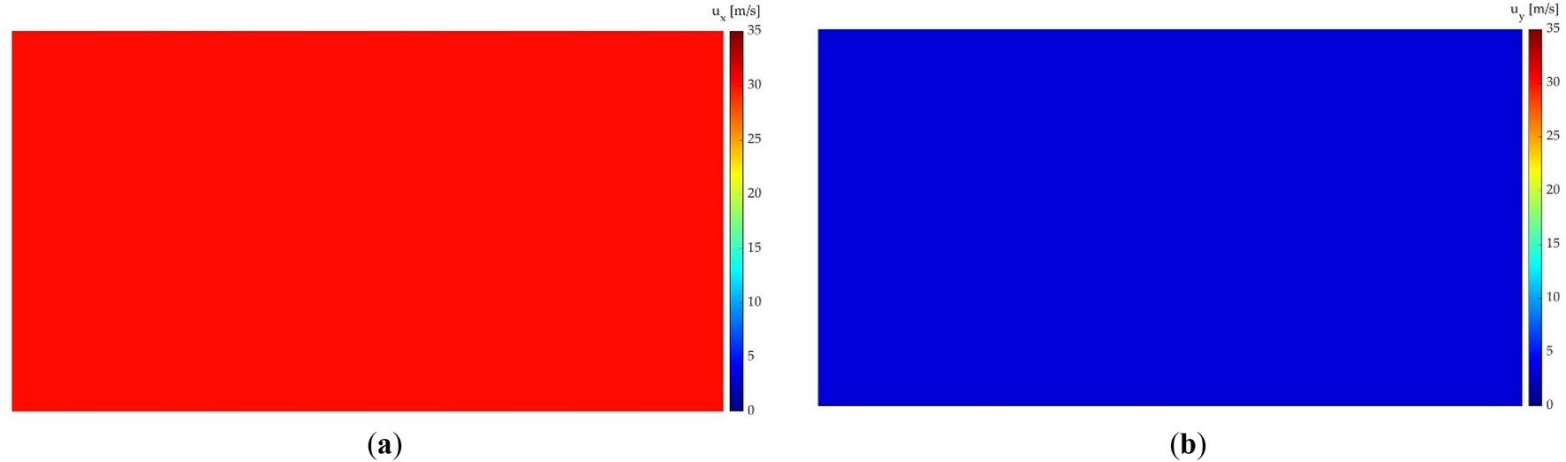
Velocity component layers: (**a**) component in x direction; (**b**) component in y direction.

The velocity layers provide the value of the velocity components. These layers are used to determine the AoA of the airfoil accurately, since in the above-explained binary-representation layers, slight variations in orientation may not be correctly represented. Figure 5a,b show the velocity layers, which represent the x and y components of the velocity, respectively.

Concerning the output of the networks, three layers and two scalars are considered. The three layers correspond to the velocity (both components) and pressure fields on the TE of the airfoil, and the scalars are the ${C}_{D}$ and ${C}_{L}$ coefficients.

To prepare the output layers, the values were first interpolated to fit into a 128 $\times$ 256 arrays. Then, the values of those arrays were normalized, following Expressions (1), (2) and (3).

1 $${u}_{x}^{*}=\frac{{u}_{x}}{{u}_{\infty }}$$

2 $${u}_{y}^{*}=\frac{{u}_{y}}{{u}_{\infty }}$$

3 $${p}^{*}=\frac{p}{\rho \cdot {u}_{\infty }^{2}}$$

where ${u}_{x}^{*}$, ${u}_{y}^{*}$ and ${p}^{*}$ are the dimensionless variables.

Finally, all the values of the layers are ranged between 0 and 1 following Expression (4), in order to speed up and enhance the training process.

4 $${\Phi }^{\mathrm{^{\prime}}}=\frac{\Phi -\mathrm{min}(\Phi )}{\mathrm{max}\left(\Phi \right)-\mathrm{min}(\Phi )}$$

where Φ is replaced by each dimensionless variable.

This last step is also followed to range between 0 and 1 the input layers and the output scalars corresponding to the coefficients.

#### CNN architecture

In the present paper, two different CNN are considered, one for velocity and pressure field prediction, and another one for drag and lift coefficient prediction. This networks were designed and trained using MATLAB 2021a^29^ commercial code with its Deep Learning Toolbox^30^.

**Figure 6:**
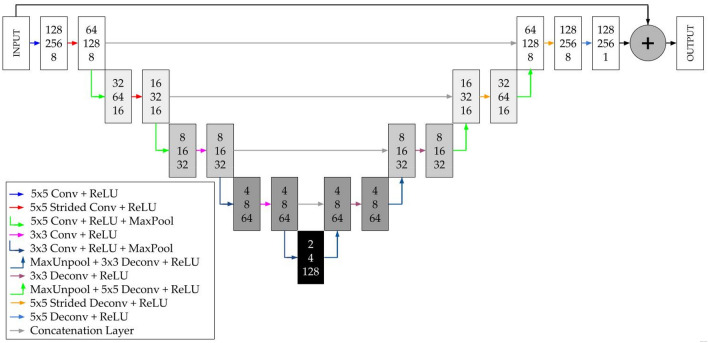
Schematic view of the proposed CNN for velocity and pressure field prediction.

For velocity and pressure field prediction, an U-Net architecture^31^ is proposed, based on the previous works from Ribeiro et al.^13^ and Thuerey et al.^18^. The U-Net architecture is a special case of an encoder-decoder network. The proposed network consists of four encoder/decoder blocks. Each encoder block contains two convolutional layers. The first one is followed by a ReLU (Rectifier Linear Unit) layer, and the second one is followed by a ReLU layer and a Max Pooling layer. The kernel size of the first two encoding blocks is equal to 5, and strided convolutions are performed on those blocks, in order to reduce the data size for the training step. The kernel size of the last blocks is equal to 3. After each encoding block, the number of filters is doubled. The decoding blocks perform the reverse process of their symmetrical blocks of the encoding phase, and they are connected to the encoding blocks by concatenation layers. Figure 6 provides a schematic view of the explained network.

**Figure 7:**
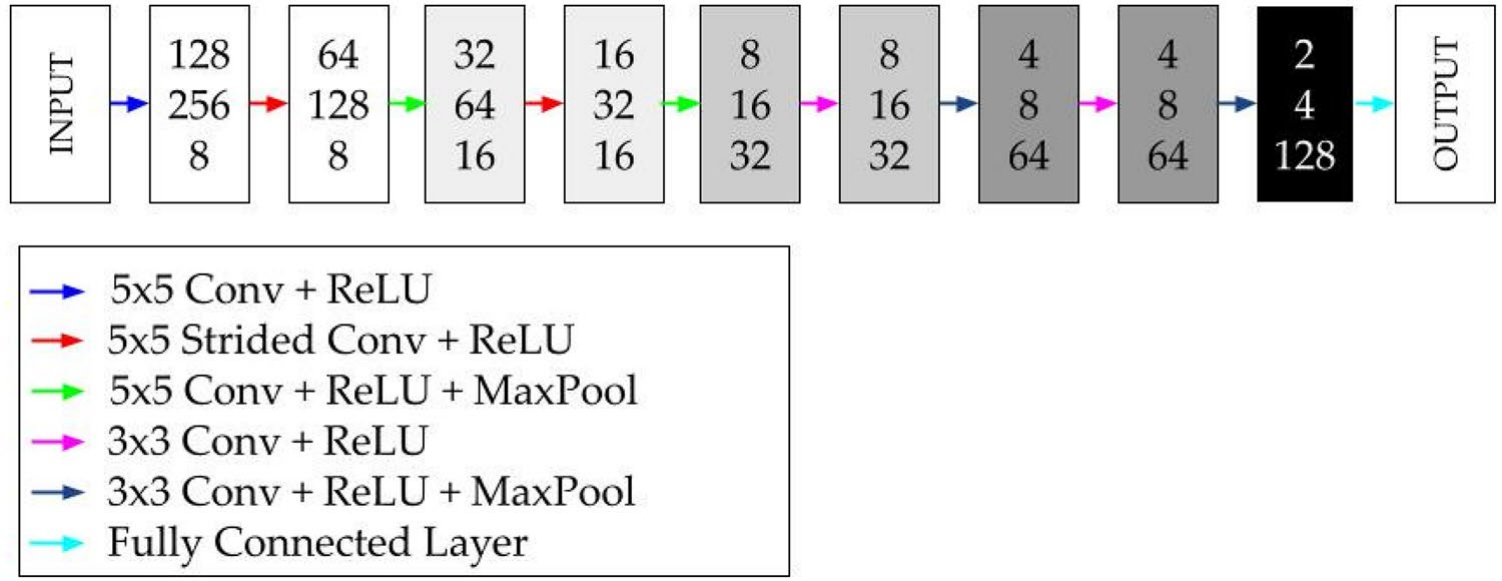
Schematic view of the proposed CNN for aerodynamic coefficient prediction.

For lift and drag coefficient prediction, only the encoding part of the CNN is considered. In this case, a fully connected layer is added after the last layer. Unlike the complete structure, this network does not return a layer, it returns two scalar values, ${C}_{D}$ and ${C}_{L}$. Figure 7 shows a schematic view of this network.

For the network training, Adam^32^ optimizer is employed in both cases, with a batch size of 64. For field prediction, a learning rate of 0.001 and a weight decay of 0.0005 is selected; and for coefficient prediction a learning rate of 0.0001 and a weight decay of 0.0005. From the dataset of 158 samples, 21 are considered for testing the network. The other 137 are divided into 70% training and 30% validation for field prediction and 90% training and 10% validation for coefficient prediction.

These hyperparameters and data-splitting ratios were selected after training this network with 27 different configurations of these parameters. From the analyzed configurations, the selected ones provide the minimum Root-Mean-Square Error (RMSE) of the analyzed magnitude. Appendix A provides a summary of the considered configurations and the obtained RMSE for each magnitude and coefficient.

## Results and discussion

In order to determine the accuracy of the proposed CNN, the predictions of this CNN have been compared with those obtained by CFD. For this comparison, the 21 simulations of the test-set mentioned above are considered.

### Velocity and pressure field prediction

**Figure 8:**
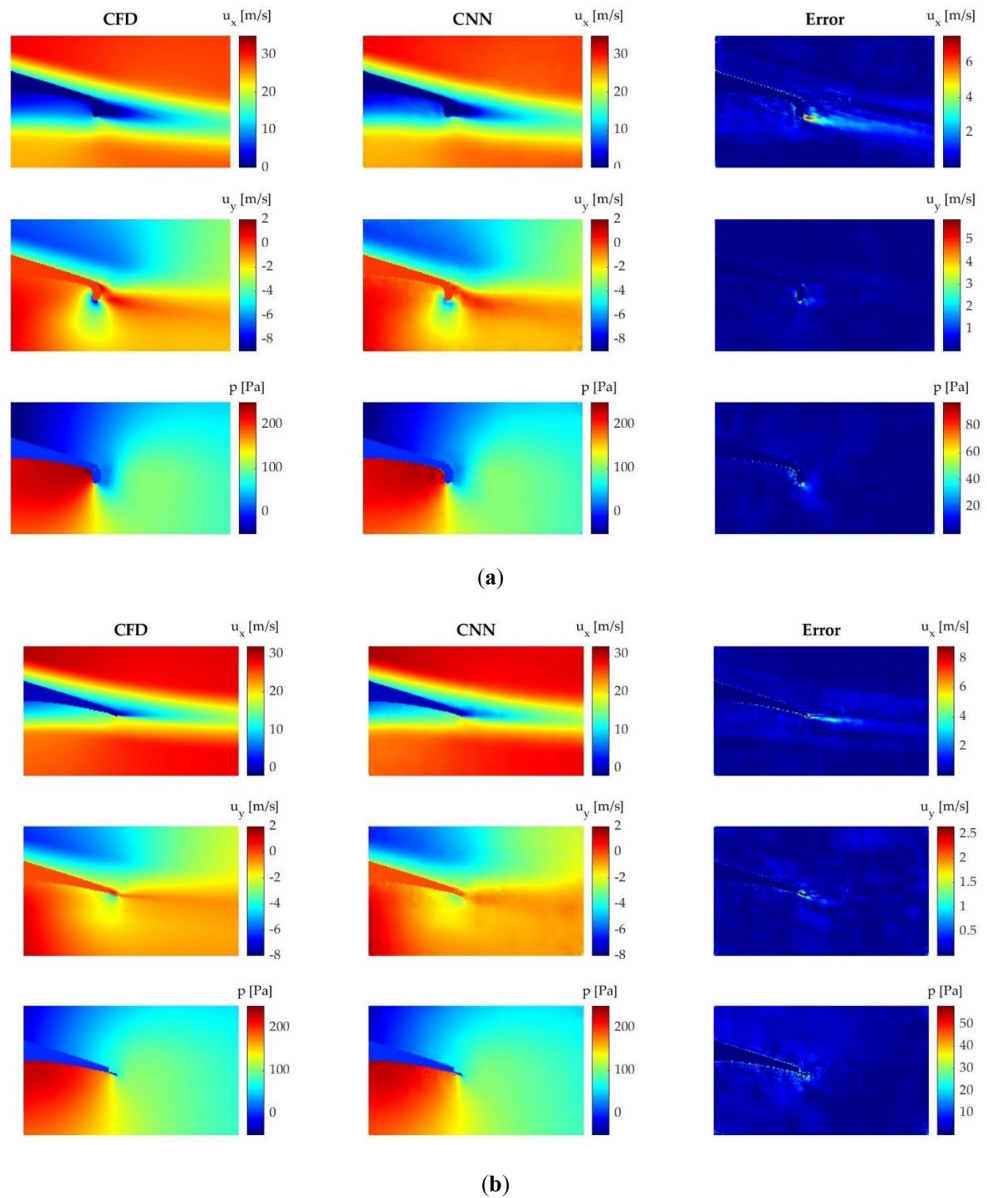

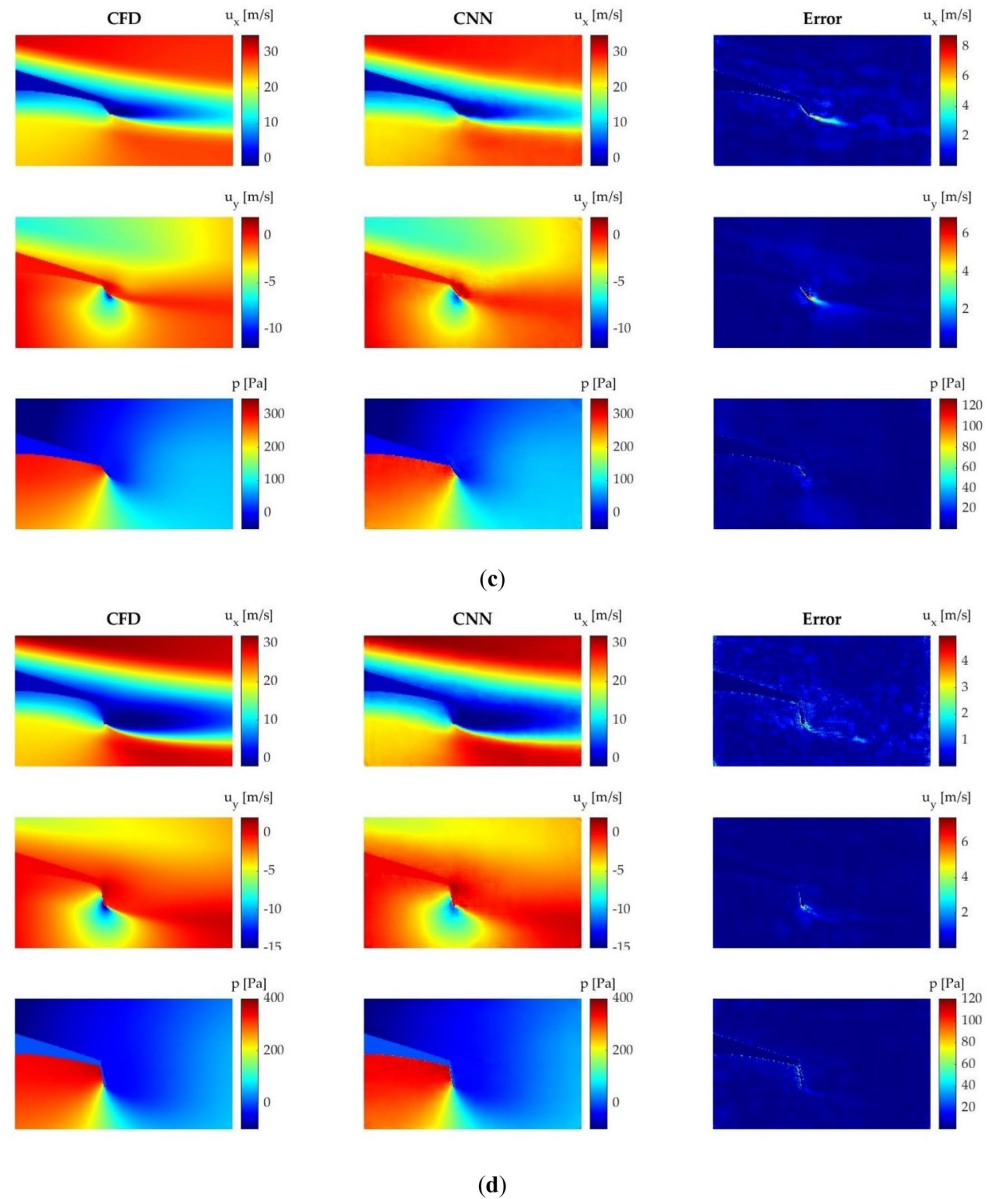
Comparison of the velocity and pressure fields obtained by means of CFD and CNN: (**a**) Gurney flap L = 1% and AoA = 0°; (**b**) microtab L = 1%, $\beta$ = − 15° and AoA = 2°; (**c**) microtab L = 1.5%, $\beta$ = − 45° and AoA = 6°; (**d**) microtab L = 2%, $\beta$ = − 75° and AoA = 9°.

The velocity and pressure fields obtained by the CNN are both qualitatively and quantitatively compared in order to determine the accuracy of the proposed CNN for field prediction. For the qualitative comparison, four different cases are considered, each one with a different geometry and AoA. All these cases can be found in Fig. 8.

The results show that the proposed CNN is able to accurately predict the velocity and pressure fields around the flow control devices in all the tested cases. The most problematic area is the wake behind the flow control device in all the analyzed cases, especially when predicting ${u}_{x}$. Some errors are also visible in the contour of the airfoil. In geometries which have surfaces perpendicular to the flow, i.e., Gurney flaps and rotating microtabs with high angles of orientation, slight errors of velocity fields appear at the front side of the flow control device. However, the CNN is able to reliably predict the flow characteristics, and all these mentioned errors are not considered significant.

**Figure 9:**
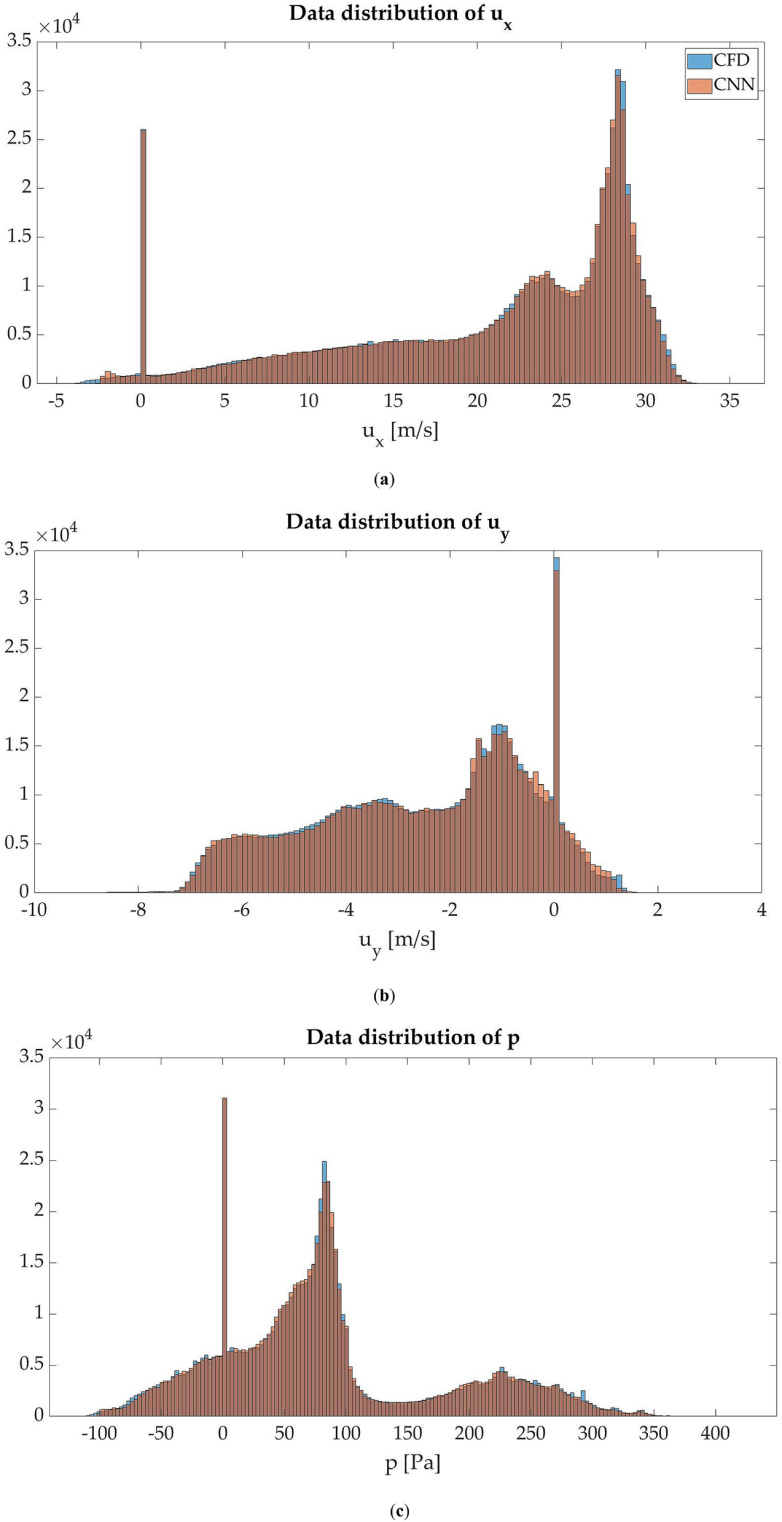
Data distribution histogram of the test-set: (**a**) data distribution of ${u}_{x}$; (**b**) data distribution of ${u}_{y}$; (**c**) data distribution of $p$.

In order to obtain a quantitative view of these results, data distribution histograms are made for each analyzed magnitude. In agreement with the qualitatively compared fields, the data distribution histograms show nearly equal shapes, being the ranges with the most data the only ones where differences between the two methods can be appreciated. Figure 9 shows data distribution histograms.

In addition, the arithmetic mean (µ) and standard deviation (σ) of both methods are calculated from the data distribution histograms. These two values, in accordance with all the results shown above, show almost equal values for all magnitudes, as shown in Table 3.

**Table 3:** Arithmetic mean and standard deviation of the results obtained by CFD and CNN.

Method	CFD			CNN		
	${u}_{x}$	${u}_{y}$	$p$	${u}_{x}$	${u}_{y}$	$p$
Arithmetic mean (µ)	20.9538	−2.5385	82.8164	20.9784	−2.5076	82.9625
Standard deviation (σ)	8.7620	2.0860	91.6118	8.7086	2.0939	90.7867

### Aerodynamic coefficient prediction

**Figure 10:**
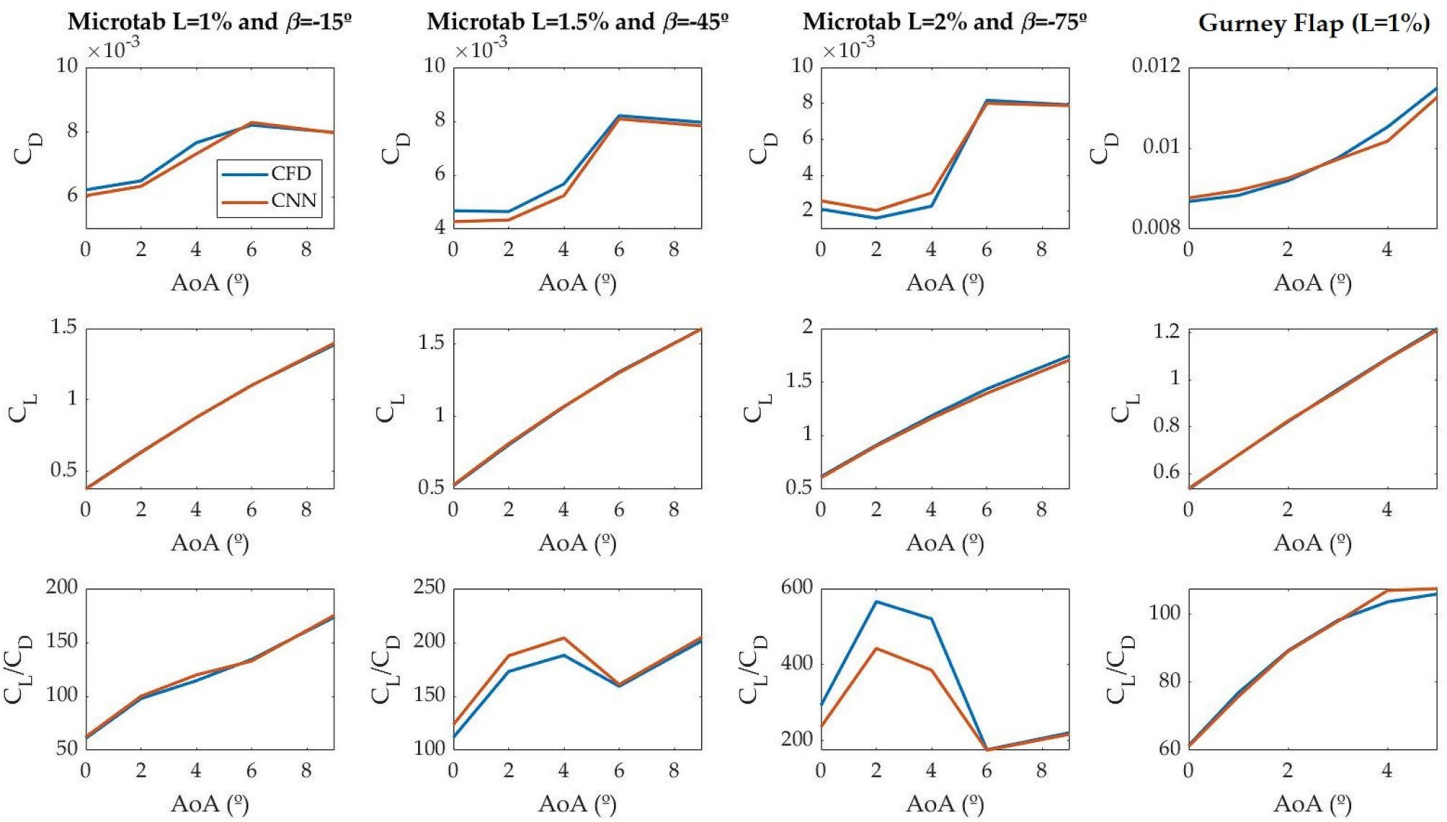
Aerodynamic coefficient comparison of all the tested cases.

In order to evaluate the accuracy of the network for predicting aerodynamic coefficients, the predicted ${C}_{D}$ and ${C}_{L}$ coefficients are compared to the benchmark values obtained by CFD simulations. In addition, the lift-to-drag (${C}_{L}/{C}_{D}$) coefficient, which is calculated from the predicted coefficients, is also compared. The plots from Fig. 10 provide this comparison.

As the plots demonstrate, the CNN is able to reliably predict aerodynamic coefficients. In the case of microtabs, ${C}_{D}$ values increase exponentially with low AoAs, and subsequently, for AoA > 6°, they decrease slightly. In the case of the Gurney flaps, this upward trend can also be seen throughout the analyzed range. In contrast, ${C}_{L}$ values follow a linear trend with respect to AoA in all the cases considered. With microtabs, the ${C}_{D}/{C}_{L}$ ratio shows a sharp rise for low AoA, followed by a less pronounced drop and a further stabilization for high AoA. The rise and the fall are more pronounced as the length of the microtab increases. When using Gurney flaps the ${C}_{D}/{C}_{L}$ coefficient follows a logarithmic trend, tending to flatten out for AoA > 4°.

${C}_{D}$ predictions show small discrepancies between the two methods. With microtabs these differences become more noticeable as the microtab length increases and the AoA decreases, and with Gurney flaps as the AoA increases. In contrast, the predictions of the ${C}_{L}$ are almost the same in all the studied cases. Nevertheless, the results show that the network is able to predict this trend, with values close to those obtained by CFD.

In order to quantify the results, the absolute and relative errors of the predictions are studied. These errors are shown in Table 4. As previously demonstrated, the errors of the ${C}_{D}$ predictions are higher than those of ${C}_{L}$, with a maximum relative error of 32.44%. However, the average relative error is 6.17%, which is considered acceptable. Low errors are observed in all the ${C}_{L}$ predictions, with an average relative error of 0.827%.

**Table 4:** Summary of absolute and relative error of ${C}_{D}$ and ${C}_{L}$ coefficients predicted by the CNN.

Error	${C}_{D}$			${C}_{L}$		
	Min	Max	Mean	Min	Max	Mean
Absolute error	0.00002	0.00074	0.00023	0.0005	0.04	0.0089
Relative error	0.19%	32.44%	6.17%	0.033%	2.786%	0.827%

### Performance analysis

The main objective of using neural networks to predict flows is to reduce the computational time required to run CFD simulations. Therefore, the computational time requirements for each method are compared. As shown in Table 5, neural networks clearly outperform CFD simulations in terms of computational time. As expected due to its simplicity, the CNN for aerodynamic coefficient prediction is the fastest one, being 16,148 times faster than the CFD simulations. However, the complete CNN used for field prediction is also considerably fast, being 7529 times faster than the CFD. Regarding the network training time, it took 12 h to train the CNN for field prediction and 5 h to train the CNN for coefficient prediction, which means a total of 17 h. This is slightly longer than the time required to perform a single CFD simulation. A single core of an Intel Xeon 5420 CPU was used for running CFD simulations and CNNs.

**Table 5:** Computational time requirement comparison.

Method	Computational time (s)	Speedup	Training time (h)
CFD	53,612	–	–
CNN (field prediction)	7.12	7529	12
CNN (coefficient prediction)	3.32	16,148	5
CNN (total)	10.44	5135	17

## Conclusions

In the present work, the implementation of flow control elements in airfoils using deep learning techniques is analyzed. With that objective, two different CNNs are proposed. One of them predicts the velocity and pressure fields around flow control devices implemented in the TE of the DU91W(2)250 airfoil, and the other one predicts the ${C}_{D}$ and ${C}_{L}$ aerodynamic coefficients of the airfoil for the same cases. These networks were trained and evaluated using the results obtained from CFD simulations, in which the cell-set model was used to implement the flow control devices. The dataset contains a total of 158 cases, with two different flow control devices, rotating microtabs and Gurney flaps, with different geometries and under different conditions.

Regarding the CNN for field prediction, the results indicate that the proposed network is able to predict the main flow characteristics around the flow control device, with very low errors, which mainly appear on the wake behind the flow control device and on the contour of the airfoil. This is attributed to the fact that the area of the wake behind the flow control device is the area in which the most differences appear between samples. Therefore, this area is the most conflicting one for the learning process of the neural network, and consequently, the area where most errors appear. Nevertheless, the network is able to correctly predict the main flow characteristics in this region.

With respect to aerodynamic coefficients, the CNN is also able to predict them accurately, with mean relative errors of 6.17% for ${C}_{D}$ and 0.827% for ${C}_{L}$. In both cases, the networks are sensitive to small changes of the geometry or the AoA, which is a key feature for geometry optimization.

In terms of computational time, the proposed networks clearly outperform the CFD simulations, reducing the computational time in four orders of magnitude.

Therefore, this paper demonstrates that flow control devices can be studied by means of neural networks, with acceptable errors and a significative reduction of required computational time and resources.

## Supplementary Information

Supplementary Information.

## Abbreviations

AI: Artificial intelligence
ANN: Artificial neural network
CFD: Computational fluid dynamics
CNN: Convolutional neural network
DL: Deep learning
HAWT: Horizontal axis wind turbine
RANS: Reynolds-averaged Navier–Stokes
ReLU: Rectifier linear unit
RMSE: Root-mean-square error
SST: Shear stress transport
TE: Trailing edge
VG: Vortex generator

## Symbols

*: Dimensionless variable
‘: Variable ranged between 0 and 1
AoA: Angle of attack
β: Flow control device orientation
$c$: Airfoil chord length
$${C}_{D}$$: Drag coefficient
$${C}_{L}$$: Lift coefficient
${C}_{L}/{C}_{D}$: Lift-to-drag ratio
$\Delta z$: First cell height
L: Flow control device length
$$\rho $$: Density
*p*: Order or accuracy (Richardson extrapolation)
$$R$$: O-mesh radius
R: Convergence condition (Richardson extrapolation)
Re: Reynolds number
RE: Richardson extrapolation solution
$$\mu $$: Dynamic viscosity
$${U}_{\infty }$$: Freestream velocity
