# Supplementary material for: CNN-based flow control device modelling on aerodynamic airfoils
Source: Sci Rep. 2022 May 17;12:8205. doi: 10.1038/s41598-022-12157-w (PMC9114417; doi:10.1038/s41598-022-12157-w)
Supplement: Supplementary file 1 — Supplementary Information. [file 41598_2022_12157_MOESM1_ESM.docx]

**Appendix A**

**Table A1.** Training RMSE of each variable with different network configurations. Network selected for field prediction highlighted in blue and network selected for coefficient prediction highlighted in green.

| **Data-split** | | **Hyperparameters** | | **RMSE** | | | | |
| --- | --- | --- | --- | --- | --- | --- | --- | --- |
| **Training Data** | **Validation Data** | **Learning Rate** | **Weight Decay** | $\boldsymbol{u}_{\boldsymbol{x}}$ | $\boldsymbol{u}_{\boldsymbol{y}}$ | $\boldsymbol{p}$ | $\boldsymbol{C}_{\boldsymbol{D}}$ | $\boldsymbol{C}_{\boldsymbol{L}}$ |
| 90% | 10% | 0.001 | 0.05 | 2.9613 | 1.8013 | 1.5827 | 0.1982 | 0.0836 |
| 90% | 10% | 0.001 | 0.005 | 2.0307 | 1.5928 | 1.1548 | 0.0754 | 0.0747 |
| 90% | 10% | 0.001 | 0.0005 | 2.5876 | 1.4668 | 1.3800 | 0.0122 | 0.0114 |
| 90% | 10% | 0.0001 | 0.05 | 4.7104 | 3.5769 | 2.7969 | 0.2127 | 0.0861 |
| 90% | 10% | 0.0001 | 0.005 | 4.5316 | 3.0442 | 2.7098 | 0.0820 | 0.0740 |
| 90% | 10% | 0.0001 | 0.0005 | 4.6642 | 2.8800 | 2.7243 | 0.0129 | 0.0062 |
| 90% | 10% | 0.00001 | 0.05 | 12.5494 | 9.6142 | 8.7675 | 0.0170 | 0.0722 |
| 90% | 10% | 0.00001 | 0.005 | 12.8985 | 10.0305 | 7.8010 | 0.0920 | 0.0766 |
| 90% | 10% | 0.00001 | 0.0005 | 13.7213 | 9.0327 | 7.1871 | 0.0141 | 0.0099 |
| 80% | 20% | 0.001 | 0.05 | 1.8785 | 1.6844 | 1.4566 | 0.1852 | 0.0649 |
| 80% | 20% | 0.001 | 0.005 | 1.6263 | 1.3941 | 1.4915 | 0.0457 | 0.0653 |
| 80% | 20% | 0.001 | 0.0005 | 1.7388 | 1.6837 | 1.3873 | 0.0653 | 0.0098 |
| 80% | 20% | 0.0001 | 0.05 | 5.1861 | 3.4236 | 2.5437 | 0.2041 | 0.0754 |
| 80% | 20% | 0.0001 | 0.005 | 4.2913 | 2.8553 | 2.6483 | 0.0434 | 0.0630 |
| 80% | 20% | 0.0001 | 0.0005 | 5.2063 | 3.2808 | 2.3179 | 0.0121 | 0.0095 |
| 80% | 20% | 0.00001 | 0.05 | 13.5421 | 8.5560 | 8.7065 | 0.2012 | 0.0860 |
| 80% | 20% | 0.00001 | 0.005 | 13.0439 | 11.4870 | 7.6283 | 0.0922 | 0.0558 |
| 80% | 20% | 0.00001 | 0.0005 | 13.9817 | 9.0319 | 8.7766 | 0.0171 | 0.0148 |
| 70% | 30% | 0.001 | 0.05 | 2.1842 | 1.9294 | 1.4428 | 0.1868 | 0.0774 |
| 70% | 30% | 0.001 | 0.005 | 2.9244 | 1.5598 | 1.1951 | 0.0761 | 0.0688 |
| 70% | 30% | 0.001 | 0.0005 | 1.4398 | 1.2134 | 1.1108 | 0.0131 | 0.0116 |
| 70% | 30% | 0.0001 | 0.05 | 8.9455 | 3.0005 | 3.0803 | 0.1753 | 0.0808 |
| 70% | 30% | 0.0001 | 0.005 | 4.5593 | 2.7137 | 2.5436 | 0.0308 | 0.0706 |
| 70% | 30% | 0.0001 | 0.0005 | 3.9979 | 3.2191 | 2.8679 | 0.0129 | 0.0071 |
| 70% | 30% | 0.00001 | 0.05 | 12.6345 | 9.9964 | 8.8872 | 0.1794 | 0.0911 |
| 70% | 30% | 0.00001 | 0.005 | 12.2996 | 9.9542 | 9.0241 | 0.0964 | 0.0685 |
| 70% | 30% | 0.00001 | 0.0005 | 13.5026 | 10.0548 | 9.5816 | 0.0165 | 0.0154 |
